# Methylene-tetrahydrofolate reductase contributes to allergic airway disease

**DOI:** 10.1371/journal.pone.0190916

**Published:** 2018-01-12

**Authors:** Kenneth R. Eyring, Brent S. Pedersen, Kenneth N. Maclean, Sally P. Stabler, Ivana V. Yang, David A. Schwartz

**Affiliations:** 1 Department of Medicine, School of Medicine, University of Colorado, Aurora, CO, United States of America; 2 Department of Pediatrics, School of Medicine, University of Colorado, Aurora, CO, United States of America; 3 Department of Immunology, School of Medicine, University of Colorado, Aurora, CO, United States of America; Institut de genomique, FRANCE

## Abstract

**Rationale:**

Environmental exposures strongly influence the development and progression of asthma. We have previously demonstrated that mice exposed to a diet enriched with methyl donors during vulnerable periods of fetal development can enhance the heritable risk of allergic airway disease through epigenetic changes. There is conflicting evidence on the role of folate (one of the primary methyl donors) in modifying allergic airway disease.

**Objectives:**

We hypothesized that blocking folate metabolism through the loss of methylene-tetrahydrofolate reductase (Mthfr) activity would reduce the allergic airway disease phenotype through epigenetic mechanisms.

**Methods:**

Allergic airway disease was induced in C57BL/6 and C57BL/6^*Mthfr*-/-^ mice through house dust mite (HDM) exposure. Airway inflammation and airway hyperresponsiveness (AHR) were measured between the two groups. Gene expression and methylation profiles were generated for whole lung tissue. Disease and molecular outcomes were evaluated in C57BL/6 and C57BL/6^*Mthfr*-/-^ mice supplemented with betaine.

**Measurements and main results:**

Loss of Mthfr alters single carbon metabolite levels in the lung and serum including elevated homocysteine and cystathionine and reduced methionine. HDM-treated C57BL/6^*Mthfr*-/-^ mice demonstrated significantly less airway hyperreactivity (AHR) compared to HDM-treated C57BL/6 mice. Furthermore, HDM-treated C57BL/6^*Mthfr*-/-^ mice compared to HDM-treated C57BL/6 mice have reduced whole lung lavage (WLL) cellularity, eosinophilia, and Il-4/Il-5 cytokine concentrations. Betaine supplementation reversed parts of the HDM-induced allergic airway disease that are modified by Mthfr loss. 737 genes are differentially expressed and 146 regions are differentially methylated in lung tissue from HDM-treated C57BL/6^M*thfr*-/-^ mice and HDM-treated C57BL/6 mice. Additionally, analysis of methylation/expression relationships identified 503 significant correlations.

**Conclusion:**

Collectively, these findings indicate that the loss of folate as a methyl donor is a modifier of allergic airway disease, and that epigenetic and expression changes correlate with this modification. Further investigation into the mechanisms that drive this observation is warranted.

## Introduction

The prevalence of asthma has been increasing over the past several decades [1, 2], and currently, 12.9% of the United States population have been diagnosed with asthma [3]. This increase in prevalence is likely due to environmental exposures as these changes have occurred too rapidly to be explained by genetics alone. Environmental influences play a major role in both the development and progression of asthma [4–7]. These environment-phenotype interactions are beginning to be better understood through increasing research in epigenetic modifications [8–13].

**Fig 1:**
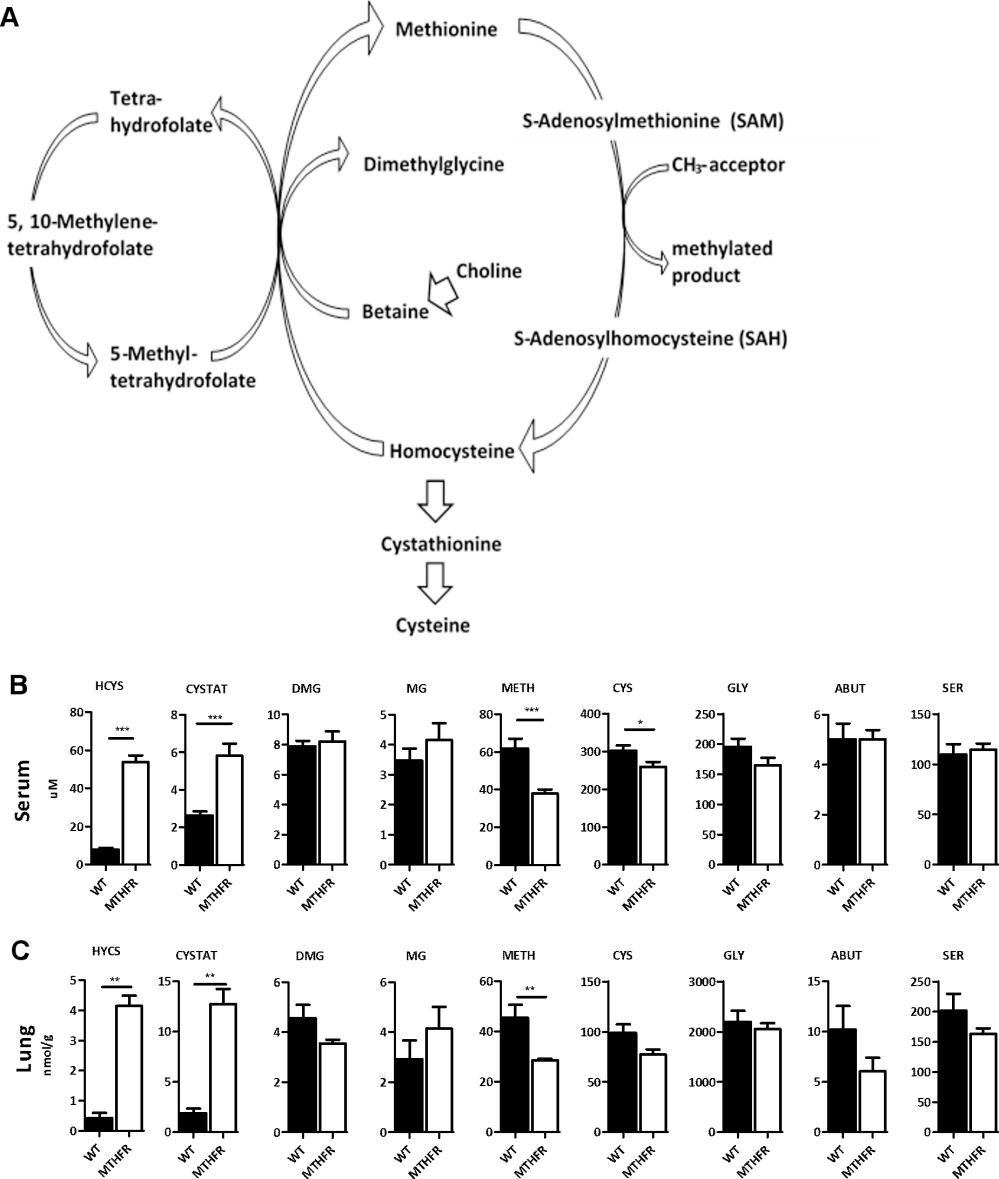
Single carbon metabolite levels differ between C57BL/6^*Mthfr*-/-^ and C57BL/6 mice. (A) Single Carbon metabolism pathway. C57BL/6^M*thfr*-/-^ mice demonstrate altered levels of single carbon metabolites (homocysteine, HCYS; cystathionine, CYSTAT; dimethylglycine, DMG; methylglycine, MG; methionine, METH; cysteine, CYS; glycine, GLY; alpha aminobutyrate, ABUT; serine, SER) in (B) serum and (C) whole lung tissue compared to C57BL/6 mice (C57BL/6 black bars; C57Bl/6^*Mthfr*-/-^ white bars, * p-value <0.05, *** p-value <0.001).

Epigenetic mechanisms are the biological processes that lead to heritable changes in gene expression [14, 15] which are important for cell differentiation, X-chromosome inactivation, genomic imprinting, and other vital cellular responses [14]. Many of these epigenetic processes are dependent on the availability of methyl groups from S-adenosyl methionine (SAM). Single carbon metabolism is the metabolic pathway that maintains SAM levels in the cell (Fig 1A). This includes the re-methylation of homocysteine to methionine with either the co-substrate 5-methyl-tetrahydrofolate or betaine. An imbalance in single carbon metabolism can lead to changes in downstream methylation [16, 17], as well as changes in allergic airway disease (unpublished data).

Methylene tetrahydrofolate reductase (MTHFR) converts 5, 10-methylene-tetrahydrofolate to 5-methyl-tetrahydrofolate which is the folate form used in homocysteine re-methylation. The most common polymorphism in *MTHFR* is the C677T SNP and homozygous TT individuals have a diminished level of global methylation compared to homozygous CC individuals [18]. While a Danish group initially found no association of *MTHFR* C677T with various outcome variables of asthma and atopic disease [19], in a subsequent larger study an increased prevalence of doctor-diagnosed asthma was observed in individuals homozygous for the minor T allele [20]. The TT genotype was also found to be associated with asthma in an Asian population [21]. In addition, children with the minor T allele and higher folate levels had an increased risk of eczema by age 4, but not wheezing or asthma [22].

While our group (unpublished data) and others [23, 24] have demonstrated a causal relationship between *in utero* exposure to methyl donors and both Th_2_ immunity and allergic airway disease in mice, the contribution of folate to allergic airway disease has yet to be determined due to the conflicting published data in human cohorts. Thus, we hypothesized that blocking folate metabolism through the loss of Mthfr activity would reduce the severity of allergic airway disease.

## Materials and methods

### Mice

C57BL/6^*Mthfr*-/-^ [25] were provided by Rima Rozen (McGill University) and were rederived at Charles River Laboratories. C57BL/6 mice were obtained through Charles River Laboratories. Animals were housed under standard conditions and protocols were approved by the Institutional Animal Care and Use Committee of the University of Colorado.

### Phenotyping

Mice were sensitized to 10 μg of filtered house dust mite extract (HDM, GREER Labs) or saline through i.p. injection on days 0 and 7 followed by challenge on days 14 and 15 with 5 μg HDM or saline administered intratracheally using a microsprayer (Penn Century). HDM was reconstituted in saline to 1 ug/ml and passed through a 0.45 μm filter then a 0.02 μm filter to facilitate microsprayer use. On day 17, mice were anesthetized by an i.p. injection of pentobarbital sodium (60 mg/kg). Following tracheostomy, pancuronium bromide (0.25 mg/kg) was administered, and mice were ventilated on a small animal ventilator (flexiVent FV-FXM1; SCIREQ). Airway resistance was measured through forced oscillation techniques (flexiVent FV-FXM1; SCIREQ) over increasing doses of methacholine (0, 12.5, 25, and 50 mg/ml).

After methacholine challenge, blood and whole lung lavage (WLL) were collected. The lavage fluid was a preparation of 50 mL of 1x phosphate buffered saline (PBS) with 60 μL of 0.5M EDTA added. Mice were lavaged with an initial 1 mL of lavage fluid followed by two consecutive lavages of 0.5 mL of fresh lavage fluid. WLLF was kept on ice throughout processing.

WLLF was then spun down and the supernatant was collected into a separate tube and stored at -80°C. The cell pellet was then treated with red blood cell lysing buffer (Sigma), spun down, supernatant removed, and resuspended in 1 mL of fresh 1x PBS. Then, cells were counted and total cells were calculated. Slides were prepared and stained with hematoxylin and eosin. After drying, 200 cells were identified and counted based on staining and morphology.

WLL supernatant was concentrated using Amicon Ultra 3K device. Total volume of the WLLF was concentrated by a factor of 3 on average.

Blood was withdrawn through cardiac puncture. It was collected in lithium heparin tubes (BD Inc.). Tubes were spun down at 1000 x g for 10 minutes. Serum was then removed and snap frozen in liquid nitrogen and stored at -80°C.

Lung tissue was then perfused with PBS and snap frozen. Cytokines in the lung lavage and IgE in the serum were measured using ELISA MAX Standard Sets and protocols from BioLegend.

### Betaine supplementation

Betaine (20g/L) was administered as previously published [26] from day -7 to day 17. Anhydrous Betaine (Sigma-Aldrich) was dissolved in drinking water (20 g/l) and was supplied ad libitum from day -7 to day 17 of the HDM sensitization and challenge protocol. Twice per week, Betaine supplemented water was restocked.

### Metabolite analysis

Homocysteine, cystathionine, dimethylglycine, methylglycine, methionine, cysteine, serine, glycine, and alpha aminobutyrate were measured in the serum and whole lung tissue through capillary stable isotope dilution gas chromatography/ mass spectrometry.

### Agilent expression array

#### Sample preparation and array processing

A minimum of 25 ng of high quality total RNA from whole lung tissue, as determined by Bioanalyzer RNA assays, was used for SurePrint G3 8x60K arrays (Agilent). One-Color Microarray-Based Gene Expression Analysis: Low Input Quick Amp Labeling protocol version 6.6 and kits were used for this expression experiment. First, RNA was incubated with Spike-in mix, T7 primer, and a cDNA master mix to generate labeled cRNA then purified using RNeasy columns (Qiagen). Then, cRNA was quantified on NanoDrop Spectrometer to determine total concentration and Cyanine 3 dye concentration. 600 ng of labeled sample was then fragmented then hybridized to the arrays for 17 hours followed by washing. Scanning was modified to meet the specifications of the NimbleGen MS200 scanner. A custom parameter on the NimbleGen MS200 scanner was developed in order to scan the Agilent arrays. Images were uploaded to Feature Extraction 11.5.11 software to extract data.

#### Analysis

Extracted features were up-loaded into Partek Genomics Suite (Partek Inc.), then underwent quantile normalization and log-2 transformation. Analysis of variance (ANOVA) was applied to determine differences between all experimental groups. Adjusted p-values were generated using the FDR q-value selection through Partek.

### Bisulfite sequencing and data analysis

To measure DNA methylation in whole lung tissue, bisulfite sequencing was performed utilizing Agilent’s SureSelect Methyl-Seq Target Enrichment System for Illumina Multiplexed Sequencing. Experimental procedures followed SureSelect Human Methyl-Seq Protocol Version B using SureSelect Methyl-Seq Reagent Kit and Mouse Methyl-Seq Capture Library. 4 μg of starting DNA was used for the assay. A final DNA concentration of 2nM per library was submitted for sequencing on the Illumina HiSeq. Each sample was sequenced twice.

Bisulfite-sequencing reads (S1 Table) were trimmed and aligned to the mouse genome *mm10* using *bwa-meth* [27] which also tabulated percent methylation at each CpG motif. Correlating sets of adjacent CpG sites were clustered together using the Adjacent Site Clustering algorithm [28]. Each cluster was required to have a minimum of three CpG sites to constitute a cluster. Methylation clusters were analyzed using a beta regression weighted on sequence read depth, and multiple testing correction was performed using the Benjamini-Hocheberg method [29].

### Analysis of DNA methylation and gene expression relationships

A beta regression between methylation loci and expression probes was performed to determine the relationship between methylation and expression data from HDM-treated C57Bl/6^*Mthfr*-/-^ mice and HDM-treated C57BL/6 mice. Analysis was performed on 6,927 methylation clusters with an uncorrected p-value<0.05 unique to the comparison between HDM-treated C57Bl/6^*Mthfr*-/-^ mice and HDM-treated C57BL/6 mice and all expression probes within 1 Mb of a methylation cluster (54,611). We fit methylation data to expression data and adjusted for Mthfr status.

### Pyrosequencing

Differentially methylated regions (DMRs) identified through bisulfite sequencing were confirmed through pyrosequencing PCR on Qiagen’s Pyromark MD. Primers for the PCR reaction were designed using the Pyromark Assay Design software (S7 Table). Extracted DNA samples were bisulfite converted using Zymo EZ DNA Methylation-Gold kits and protocol. Unmethylated and methylated controls were included for each primer set. Methylation measurements were averaged across duplicates then across experimental group.

### Quantitative RT-PCR

Differential expression was confirmed through qRT-PCR using TaqMan assays (Life Technologies). RNA was converted to cDNA using Superscript III Reverse Transcriptase (Life Technologies) kit and protocol. cDNA was then mixed with TaqMan Fast Advanced Master Mix and best coverage TaqMan assays then run in triplicate on the ViiA 7 Real-Time machine. Delta CT values were generated from the reference gene, beta-actin.

### Statistics

Data were expressed as mean ±SEM. Individual comparisons between groups were confirmed by a 2-tailed Mann-Whitney U test in all cases except for betaine supplementation experiments, where a 1-tailed test was used because we were testing only 1 outcome, that betaine restores the phenotype. Pyrosequecning and qRT-PCR data were analyzed using a 1-tailed Mann-Whitney U test. Significant differences between groups were identified by analysis of variance. GraphPad Prism version 5.04 (GraphPad Software, La Jolla, CA) was used to perform statistical calculations. Pathway analysis was performed using Ingenuity Pathway Analysis (IPA) software. Data deposited in the GEO database (GS71823, http://www.ncbi.nlm.nih.gov/geo/query/acc.cgi?token=odetoocutfgvjyd&acc=GSE71823).

## Results

### Loss of Mthfr activity altered metabolite levels in single carbon metabolism

Loss of Mthfr activity altered single carbon metabolite concentrations (Fig 1B and 1C). C57BL/6^*Mthfr*-/-^ mice showed a significant increase in serum homocysteine (HYCS) and cystathionine (CYSTAT) while methionine (METH) and cysteine (CYS) are decreased compared to C57BL/6 mice (Fig 1B). These changes in the serum corresponded with those in whole lung tissue for HYCS, CYSTAT, and METH (Fig 1C). These results are due to the loss of co-substrate 5-methyl-tetrahydrofolate hindering the re-methylation of HYCS to METH. Although betaine is a co-substrate and available in the re-methylation of homocysteine, alone it is insufficient in maintaining normal levels of HYCS and METH in the absence of 5-methyl-tetrahydrofolate. HDM treatment had no effect on metabolite levels.

### Suppressed allergic airway disease is observed in C57BL/6^*Mthfr*-/-^

**Fig 2:**
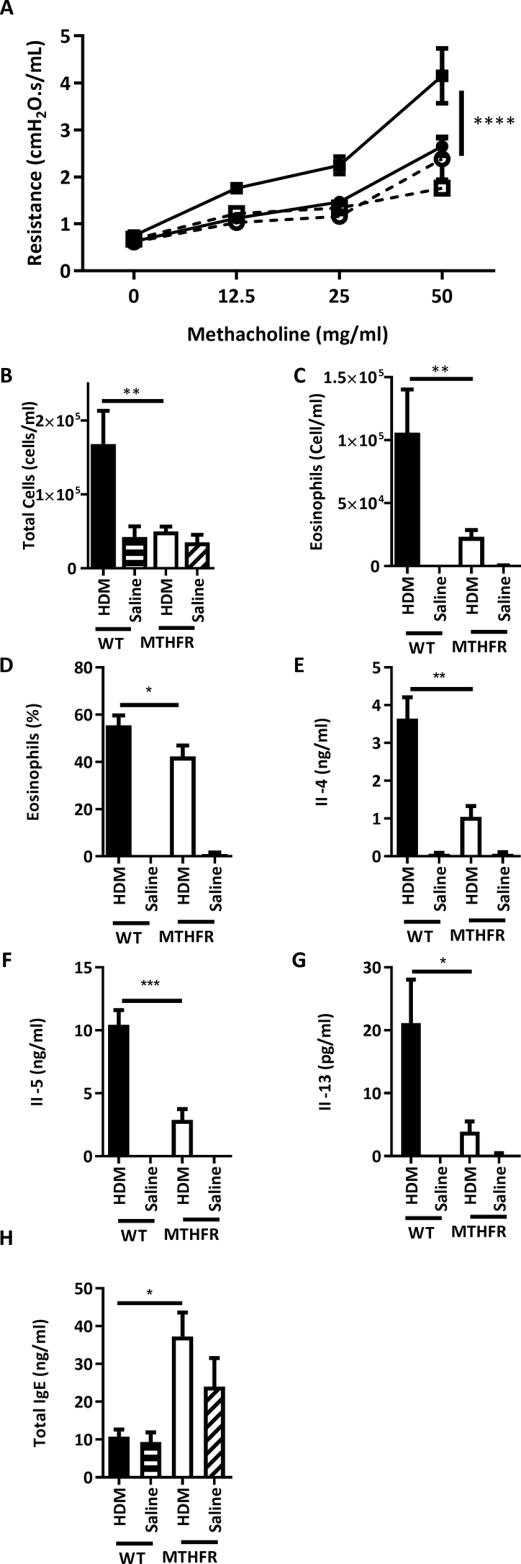
Allergic airway disease was suppressed in C57BL/6^*Mthfr*-/-^ mice compared to C57BL/6 mice. Loss of Mthfr activity suppressed the severity of HDM-induced allergic airway disease with virtually no changes at baseline. (A) airway hyperresponsiveness (HDM-treated C57BL/6 solid line closed square, HDM-treated C57BL/6^*Mthfr*-/-^ solid line closed circle, saline-treated C57BL/6 dashed line open square, and saline-treated C57BL/6^*Mthfr*-/-^ dashed line open circle), (B) total cells in WLL, and (C) concentration of eosinophils, (D) percentage of eosinophils in the WLL, (E) WLL Il-4 cytokine levels, (F) WLL Il-5 cytokine levels, (G) WLL Il-13 cytokine levels, and (H) total IgE levels in the serum (HDM-treated C57BL/6 black bar, HDM-treated C57BL/6^*Mthfr*-/-^ white bar, saline-treated C57BL/6 black bar with white dots, and saline-treated C57BL/6^*Mthfr*-/-^ white bar with black dots, * p-value <0.05, ** p-value <0.01, *** p-value <0.001).

HDM-treated C57BL/6^*Mthfr*-/-^ mice demonstrated a suppressed allergic airway disease phenotype compared to HDM-treated C57BL/6 mice including reduced AHR (Fig 2A) and decreased cellularity and eosinophilia in WLL (Fig 2B–2D). Furthermore, HDM-treated C57BL/6^*Mthfr*-/-^ mice showed a significant reduction in Il-4, Il-5, and Il-13 cytokine concentrations in the WLL compared to HDM-treated C57BL/6 mice (Fig 2E–2G). However, serum total IgE levels did not follow this pattern (Fig 2H). Interestingly, HDM-treated C57BL/6^*Mthfr*-/-^ mice had higher levels of total IgE compared to HDM-treated C57BL/6 mice (Fig 2H). Further studies are necessary to elucidate the mechanism of this finding. No statistical differences between saline-treated C57BL/6^*Mthfr*-/-^ and saline-treated C57BL/6 mice were observed for AHR, cellular recruitment, and cytokine levels suggesting that differences in disease state are not a result of a shift in baseline levels except for total IgE levels in the serum while not significant similar differences were present (Fig 2).

HDM-treated C57BL/6 mice were phenotypically different in all measurements compared to saline-treated C57BL/6 mice including an increase in AHR and cellularity, eosinophilia, and cytokine concentrations in the WLL (Fig 2). HDM-treated C57BL/6^*Mthfr*-/-^ mice only differed from the saline-treated C57BL/6^*Mthfr*-/-^ mice by the number and percentage of eosinophils in the WLL (Fig 2). Additionally, detected Ifn-γ in saline-treated mice suggests a slight Th1 bias which is skewed toward Th2 upon HDM-treatment though less extensively in HDM-treated C57BL/6^*Mthfr*-/-^ mice (S1 Fig). However, the HDM response is not skewed toward Treg or Th17 since Il-10 and Il-17 were not detected.

In summary, loss of Mthfr activity resulted in a suppression of allergic airway disease including the lack of AHR and a reduction of cellular recruitment into the airway without a shift at baseline. However, these mice still developed allergic airway disease characteristics such as increased inflammatory cytokines and eosinophil recruitment.

### Gene expression differences between C57BL/6^*Mthfr*-/-^ and C57BL/6 mice

**Fig 3:**
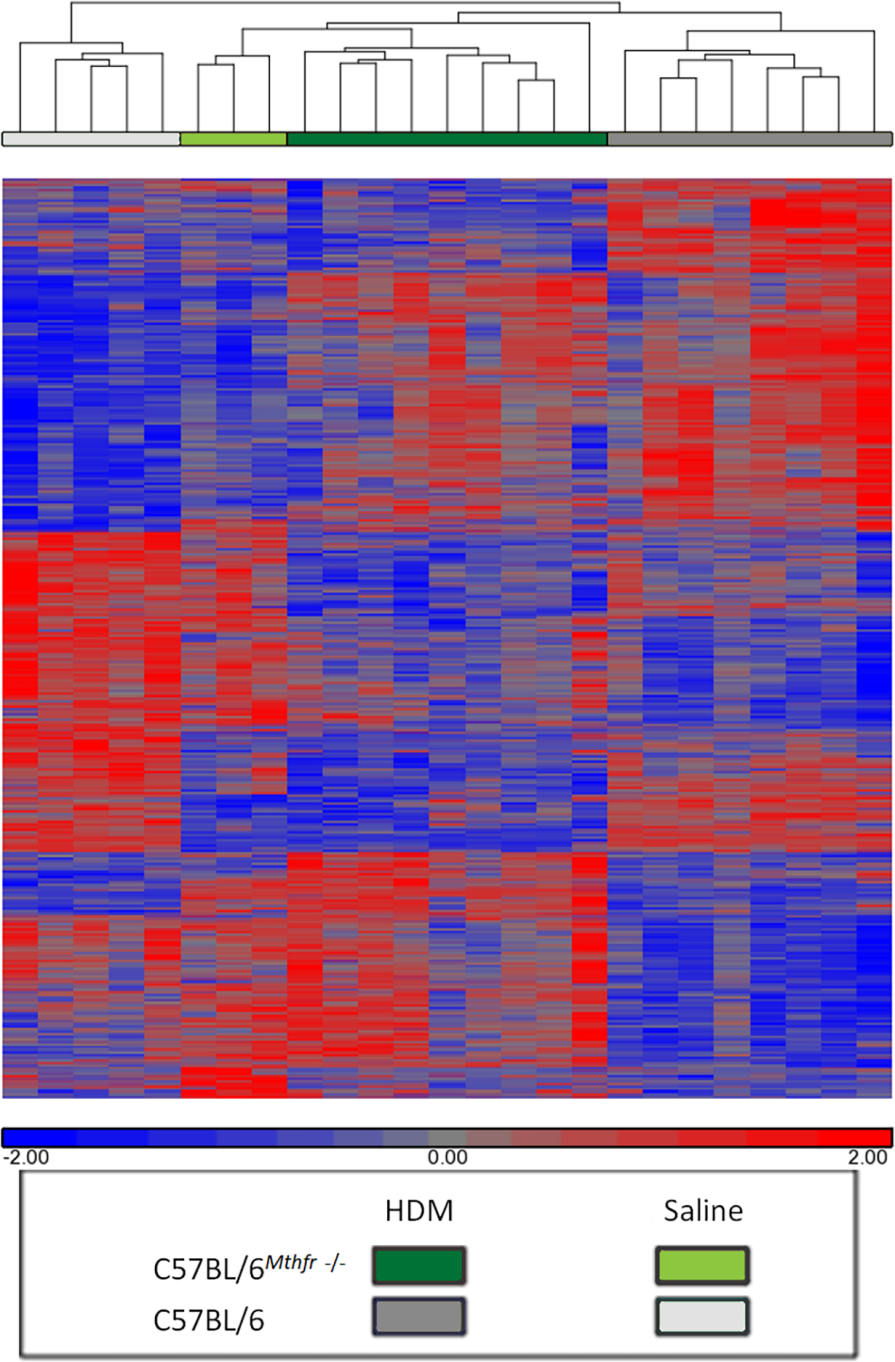
Hierarchal clustering of differentially expressed genes between HDM-treated C57BL/6^*Mthfr*-/-^ and HDM-treated C57BL/6. ANOVA analysis identified 2,588 transcripts that are differentially expressed between the four experimental groups. Gene clustering by similarity in expression as quantified by the Pearson correlation using average linkage hierarchical clustering show groups of genes related to *Mthfr* status as well as treatment dependent clusters.

ANOVA analysis identified 737 genes that are differentially expressed in HDM-treated C57BL/6^Mthfr-/-^ compared to HDM-treated C57BL/6 mice with an adjusted p-value <0.05. 30 of the 737 genes are shared in a similar comparison between saline-treated C57BL/6^*Mthfr*-/-^ and saline-treated C57BL/6 mice (36). These baseline differences were subtracted from further analyses. Gene clustering by similarity in expression as quantified by the Pearson correlation using average linkage hierarchical clustering identified treatment dependent as well as *Mthfr* status dependent expression patterns (Fig 3).

IPA identified several enriched pathways (S2 Table). As to be expected with C57Bl/6^*Mthfr*-/-^ mice, a number of biochemical pathways were enriched as loss of Mthfr shifts the balance of other folate forms [18]. There were also a number of immune signaling and developmental pathways identified (S2 Table). S2 Fig is a representative network showing the known interactions of immune genes.

### Alterations in C57BL/6^*Mthfr*-/-^ methylation

Interruption of single carbon metabolism resulted in substantial changes in DNA methylation [9, 16, 17]. Therefore, it is predicted that DNA methylation in C57BL/6^*Mthfr*-/-^ mice would be altered. Our analysis of 99Mb of the genome selected for areas of high CpG density (Agilent SureSelect) identified a cluster of DMRs around the *Mthfr* gene. Comparing sequence data to known B129 SNP data from the Mouse Genome Informatics database identified a 14Mb region on chromosome 4 in the C57BL/6^*Mthfr*-/-^ mice as B129 background (S3 Fig). Back-crossing to a pure strain does not result in a 100% congenic strain, thus these results are not unexpected. All results located in this region have been excluded from further discussion.

**Fig 4:**
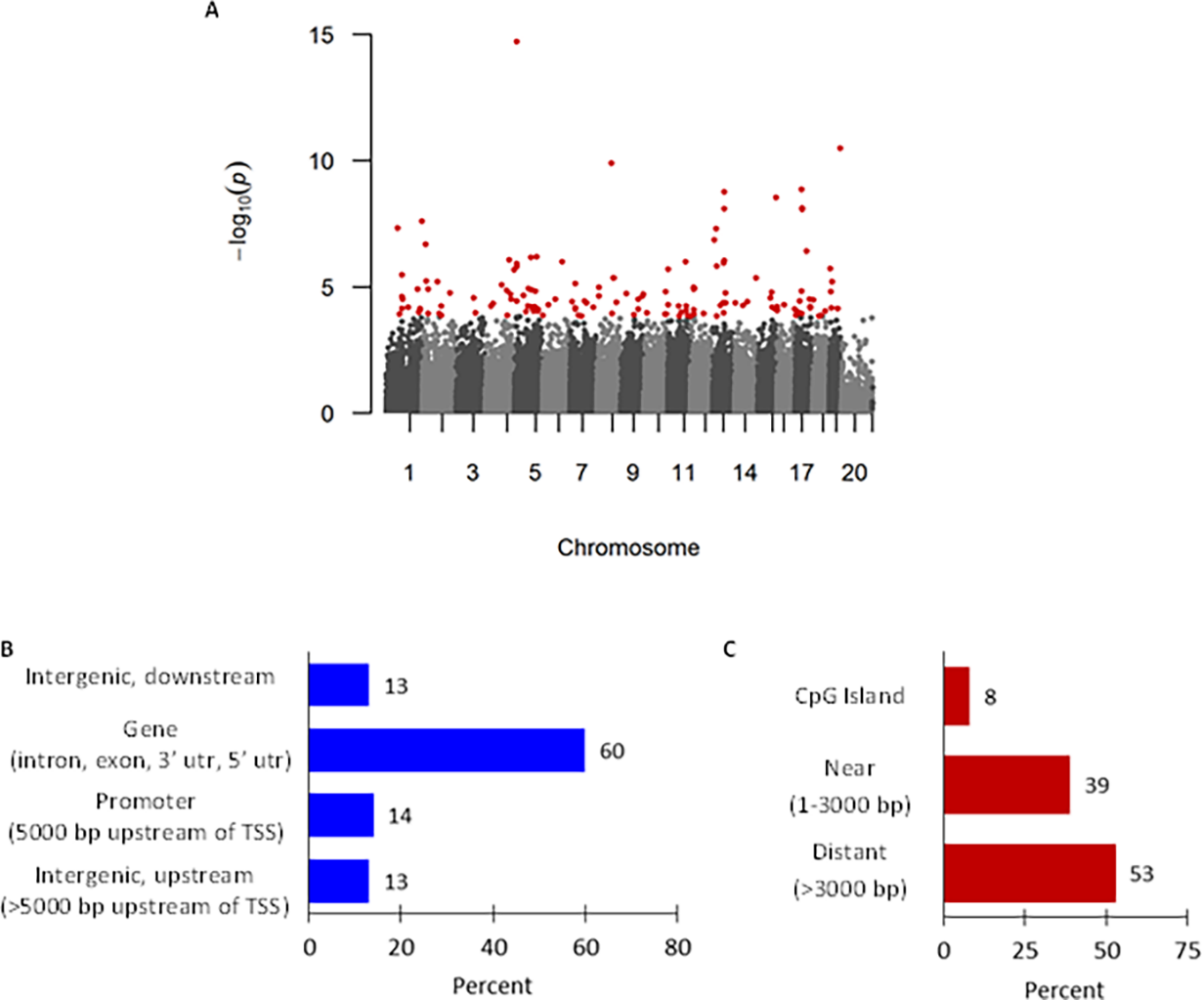
Loss of Mthfr activity in HDM-treated mice was associated with differential DNA methylation. (A) Manhattan plot of the p-values from a weighted beta regression for HDM-treated C57BL/6^*Mthfr*-/-^ vs. HDM-treated C57BL/6 mice. Each dot represents a p-value for correlating CpG clusters as identified through A-clustering with a minimum of 3 CpGs within a cluster. Red dots denote statistically significant differentially methylated regions (DMRs) after adjustment for multiple testing correction. Genomic distribution of 146 DMRs by relationship to (B) gene and (C) CpG Island.

In the HDM-treated mice, 146 DMRs with an adjusted p-value ≤0.10 were identified between C57BL/6^*Mthfr*-/-^ and C57BL/6 mice (Fig 4A). 32 of the 146 DMRs were shared in a similar comparison between saline-treated C57BL/6^*Mthfr*-/-^ and saline-treated C57BL/6 mice (247) in terms of physical location or shared nearest gene. These baseline differences were subtracted from further analyses. Of the remaining 114 DMRs (S3 Table), the median length is 90 base pairs (range, 8–719, S3 Table) and were primarily found in gene bodies (intron, exon, 3’ untranslated region, or 5’ untranslated region; n = 68 or 60%; Fig 4B) and areas distant from CpG islands (>3000 bases from the island; n = 61 or 54%; Fig 4C) with the least amount located in the promoter or within CpG islands. There was a significant enrichment of hypomethylated DMRs (n = 94, p-value = 1.2 x 10^−12^, S3 Table) in HDM-treated C57BL/6^*Mthfr*-/-^ mice.

Furthermore, we performed subset analyses on methylation clusters within 25kb of asthma related genes as defined by genetic association [30], Ingenuity Pathway Analysis, genes included in the top 10 enriched IPA expression pathways from the study, or overlap of the 3 lists (S4 Table). Statisically significant DMRs (adjusted p-value <0.10) were identified near *Tle4* and *Tnf*; *Scn5a*, *Pde7b*, *Tnf*, and *Rasgrp4*; *Nod2*; and *Tlr9* and *Tnf* respectively (S4 Fig). In summary, our methylation analysis identified that disruption of single carbon metabolism in allergic airway disease leads to a unique methylation profile enriched with hypomethylated DMRs and highlights genes previously associated with disease.

### Confirmation of expression and methylation analysis

**Fig 5:**
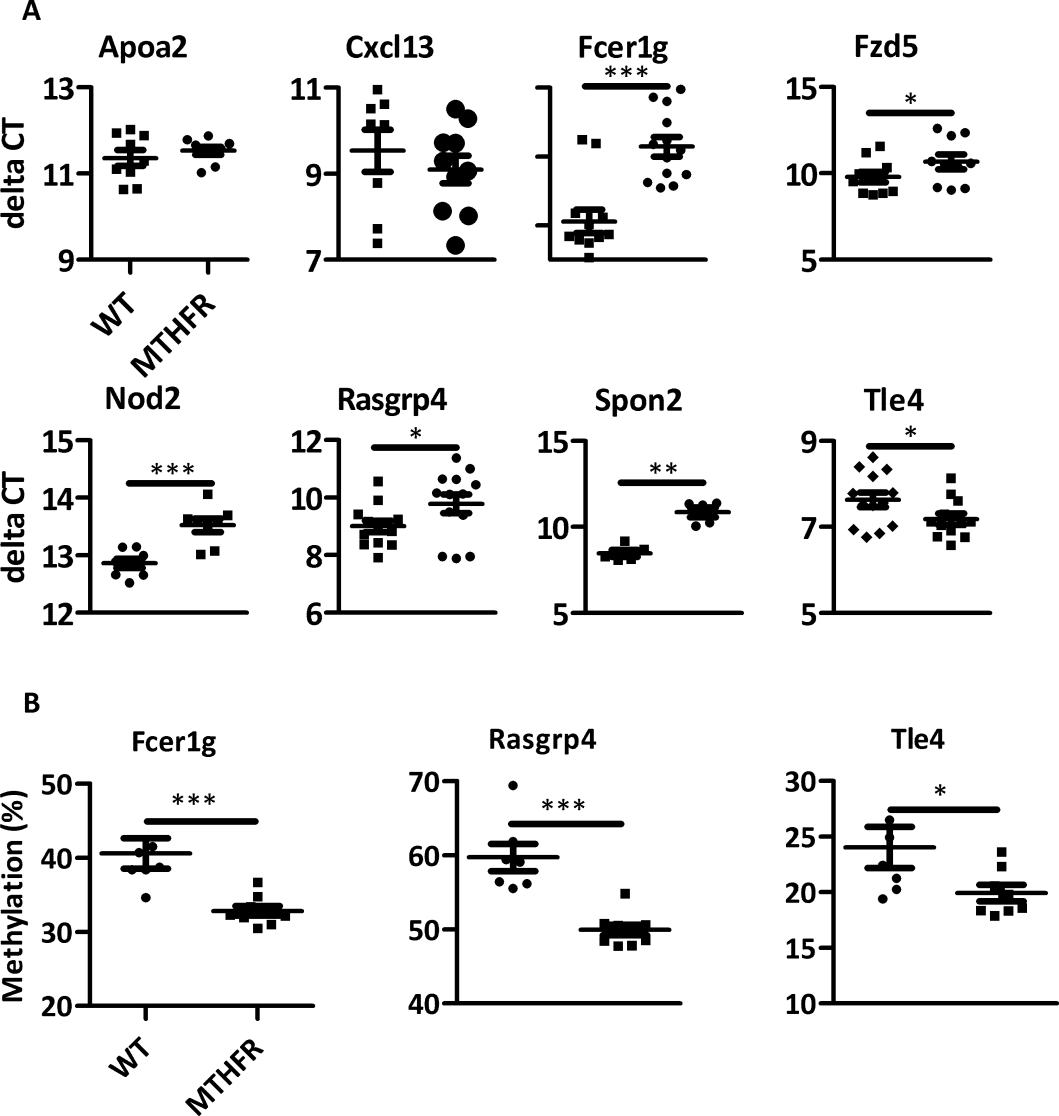
Expression and methylation results were confirmed. (A) 8 differentially expressed genes from the array data were quantified through RT-PCR. 6 out of 8 genes validated. (B) *Rasgrp4*, *Tle4*, and *Fcer1g* DMRs were validated through pyrosequencing. (* p-value <0.05, ** p-value <0.01 *** p-value <0.001).

Differentially expressed genes between HDM-treated C57Bl/6^*Mthfr*-/-^ mice and HDM-treated C57BL/6 mice from the array data were confirmed through qRT-PCR. Genes prioritized for validation were chosen based on statistical significance, fold-change, biological function, and/or proximity to a DMR (Fig 5A). Out of the 8 genes tested, changes in 6 genes were statistically significant (p-value <0.05) and had same directionality and similar magnitude of changes in expression to the array data (Fig 5A).

DMRs were prioritized for validation based on an adjusted p-value <0.10, significant expression differences of nearby genes, and biological function of those genes (Fig 5B). Within the DMRs, percent difference between experimental groups, significant t-test on individual CpGs, and the ability to design primers factored into selecting CpGs for pyrosequencing. DMRs in or near *Rasgrp4*, *Fcer1g*, and *Tle4* were tested. The DMRs validated based on their relation to the methyl sequencing data including statistical difference in the comparison of HDM-treated C57BL/6^*Mthfr*-/-^ and HDM-treated C57BL/6 mice and similar directional change in percent methylation (Fig 5B).

### Methylation-expression quantitative trait region analysis

In relation to expression, there were 7,101 expression probes within 1Mb of the statistically significant DMRs. Of these probes, there was a significant enrichment of canonical anti-correlated methylation-expression relationships (n = 4143, p-value = 1.2 x 10^−8^); additionally, there was a significant enrichment of transcripts with an uncorrected p-value <0.05 (n = 1,046, p-value = 2.2 x 10^−16^). In order to more directly measure the relationship between methylation and expression, correlation was tested between nominally significant DMRs (n = 6,927) and expression probes within 1 Mb (n = 54,611). 503 significant relationships (corrected p-value <0.05) were identified between expression and methylation data consisting of 354 unique methylation clusters and 491 expression probes (S5 Table). We observed slightly more negative correlations (273), but did not deviate from a normal distribution (p-value = 0.06, S5 Table).

**Fig 6:**
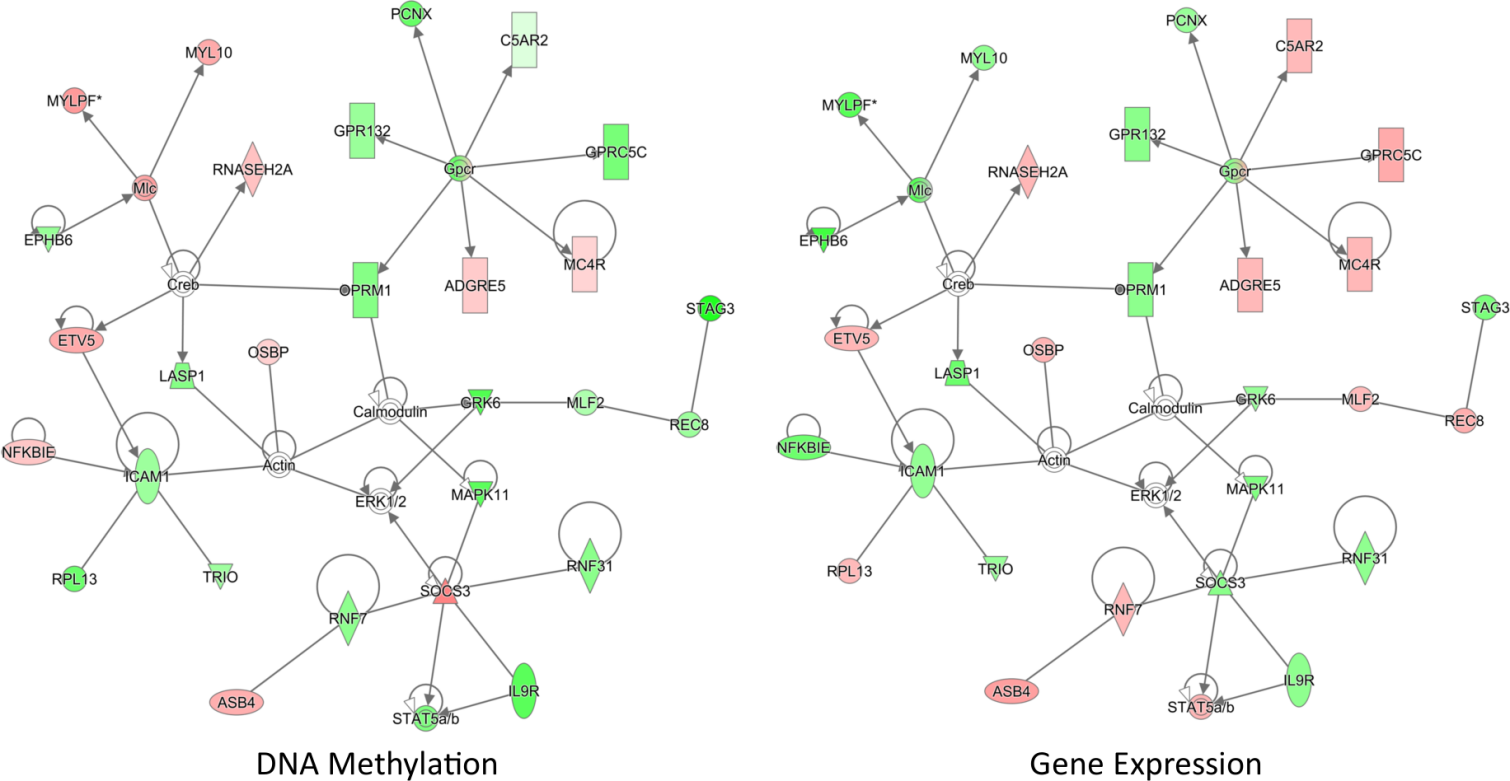
Network of methylation changes directly correlated with transcriptional activity. Methylation changes directly correlated with transcriptional activity. Methylation-expression relationships were measured by beta regression of differentially methylated regions (DMRs, uncorrected p-value <0.05, n = 6,927) associated with HDM-treated C57BL/6^*Mthfr*-/-^ mice and all expression probes found within 1Mb of each DMR. Ingenuity Pathway Analysis (IPA) on the 503 significant methylation-expression correlations (adjusted p-value <0.05) identified a significant inflammatory response network (score = 52). Green indicates lower methylation or expression and red indicates higher methylation or expression in HDM-treated C57BL/6^*Mthfr*-/-^ mice. Methylation values are colored based on relative methylation change between HDM-treated C57BL/6^*Mthfr*-/-^ and HDM-treated C57BL/6 mice; colors of expression values are based on fold change between HDM-treated C57BL/6^*Mthfr*-/-^ and HDM-treated C57BL/6 mice. Molecule shapes: horizontal oval = transcriptional regulator; vertical oval = transmembrane receptor; diamond = enzyme; up triangle = phosphatase; down triangle = kinase; trapezoid = transporter; circle = other; double circle = group; rectangle = G-protein coupled receptor. This analysis was restricted to only direct relationships. The network score is based on the hypergeometric distribution, and is calculated with the right-tailed Fisher’s exact test to identify enrichment of correlated methylated/expressed genes in the network relative to IPA database. The other network with a score greater than 40 is shown in S5 Fig.

24 pathways were enriched by genes affected by methylation changes, including many involved in inflammatory response (S6 Table). Furthermore, network analysis identified two high-scoring (score >50) networks involved in inflammatory response and development (Fig 6 and S4 Fig). The inflammatory response network contains several genes that have been previously associated with allergic disease, including *Socs3*, *Il9r*, *Icam1*, and *Stat5b*. Additionally, *Etv5* is a transcription factor known to positively regulate *Icam1* [31] and controls Th17 differentiation through the regulation of *Il17a* and *Il17f* expression [32]. These data show that specific methylation changes directly correlate with transcriptional activity of immune related genes.

### Betaine supplementation

**Fig 7:**
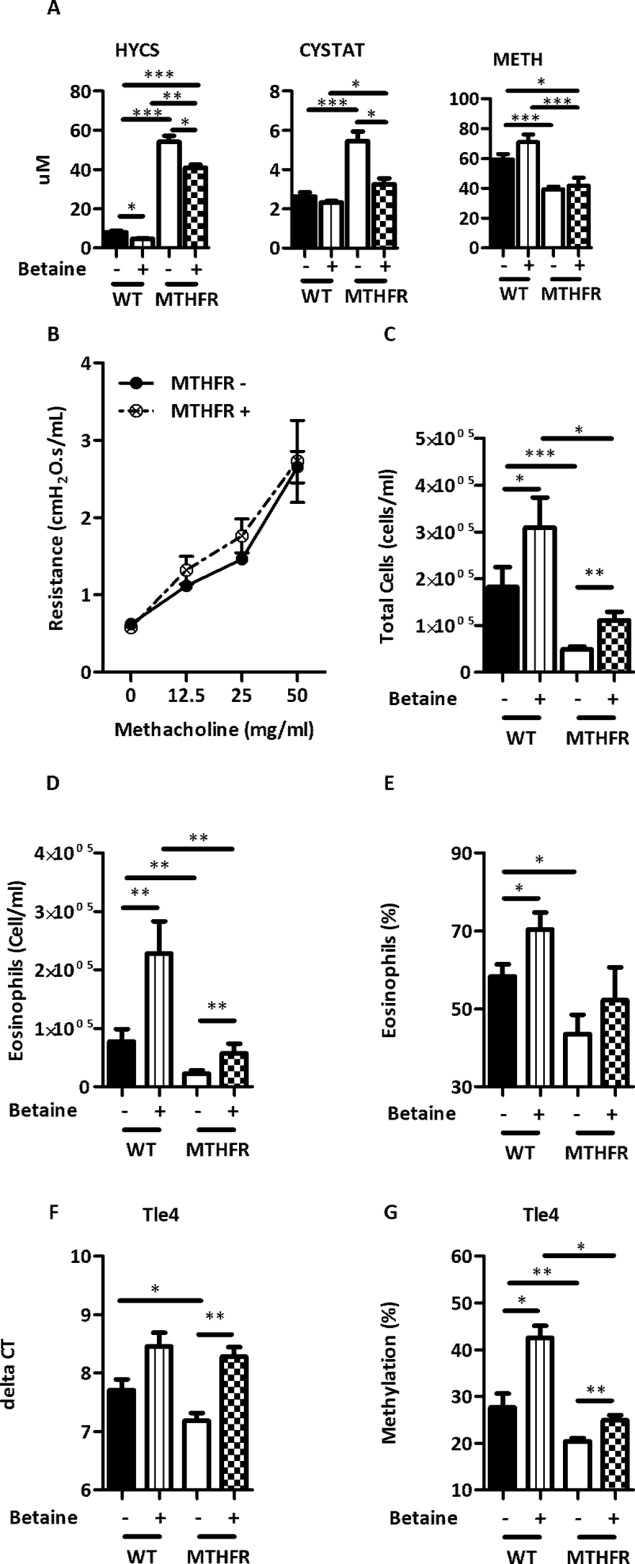
Betaine supplementation exacerbated allergic airway disease. Betaine supplementation affected single carbon metabolites (A) HYCS, CYSTAT, and METH (not supplemented C57BL/6 black bars, supplemented C57BL/6 vertical striped bars, not supplemented C57Bl/6^*Mthfr*-/-^ white bars, supplemented C57BL/6^*Mthfr*-/-^ checkered bars). Betaine supplementation exacerbated the severity of HDM-induced allergic airway disease and abolished the effect of Mthfr loss. (B) Airway hyperresponsiveness (not supplemented HDM-treated C57BL/6^*Mthfr*-/-^ solid line closed circle, and supplemented C57BL/6^*Mthfr*-/-^ dashed line open circle and X), and (C) total cells, (D) eosinophils, (E) percentage of eosinophils in WLL. (F) Expression and (G) methylation of *Tle4* is altered by betaine supplementation. (* p-value <0.05, ** p-value <0.01 *** p-value <0.001).

To confirm the role of Mthfr loss in the attenuation of allergic airway disease, C57BL/6^*Mthfr*-/-^ and C57BL/6 mice were supplemented with betaine prior to and during HDM treatment. Betaine was selected as it is another co-substrate in the re-methylation of homocysteine and can decrease HCYS to compensate for the loss of 5-methyltetrahydrofolate in the C57BL/6^*Mthfr*-/-^ mice (refer to Fig 1A). Betaine supplementation resulted in a statistically significant reduction of HCYS and CYSTAT in the serum of C57BL/6^*Mthfr*-/-^ mice without a change in METH levels (Fig 7A). C57BL/6 mice also demonstrated a decrease in HCYS, but lacked a significant change in CYSTAT or METH (Fig 7A).

Betaine supplemented HDM-treated C57BL/6^*Mthfr*-/-^ compared to HDM-treated C57BL/6^*Mthfr*-/-^ mice that were not supplemented with betaine exhibited a significant increase in cellularity and eosinophilia in the WLL without an effect on AHR (Fig 7B–7E). These modified WLL levels are similar to those of HDM-treated C57BL/6 mice that were not supplemented with betaine (Fig 7B–7E). In addition, supplementation in HDM-treated C57BL/6 mice increased the total cells and eosinophils in the WLL compared to values obtained from mice not supplemented with betaine (Fig 7C–7E). Reversing the AHR phenotype may require longer supplementation with betaine or may not be reversible as lung tissue is fairly differentiated and relatively static, and the underlying molecular drivers of the phenotype could be developmental in origin. Whereas, the immune system is still relatively undifferentiated and plastic at time of supplementation. Supplementation appeared to have no measured effect on saline-treated C57BL/6 mice (data not shown).

In addition to disease phenotype, we assessed expression and methylation levels of *Tle4*; *Tle4* had the strongest evidence for differential methylation and expression in C57BL/6^*Mthfr*-/-^ mice treated with HDM. Betaine supplementation significantly reduced the expression of *Tle4* in HDM-treated C57BL/6^*Mthfr*-/-^ mice with a significant increase in DNA methylation. Again, supplementation restored these values to levels comparable to that of HDM-treated C57BL/6 mice not supplemented with betaine. Similarly, HDM-treated C57BL/6 mice demonstrated a statistically significant increase in DNA methylation and a near significant decrease (p-value = 0.06) in expression upon betaine supplementation compared to mice not supplemented with betaine (Fig 7F and 7G).

In summary, the suppressive effect of Mthfr loss was rescued through betaine supplementation and generated a disease phenotype similar to HDM-treated C57BL/6 mice. Moreover, the specific C57BL/6^*Mthfr*-/-^ expression and methylation pattern of *Tle4* is reversed through supplementation.

## Discussion

This study demonstrates that Mthfr and single carbon metabolism may play a role in allergic airway disease. Loss of Mthfr further highlights that disruption of single carbon metabolism leads to molecular changes that reduce Th2 immune responsiveness and allergic airway disease in mice; moreover, we reversed this phenotype through betaine supplementation. These specific findings provide additional understanding of gene environment interactions and epigenetically controlled genes that are relevant in allergic airway disease.

Early immune development is altered in children affected by asthma and allergic disease. This alteration in immune development is characterized by a deficiency of IFNγ production [33, 34], altered innate immunity [35], and deficient Treg networks [36] ultimately leading to uncontrolled Th2 immune responses. Disruption of single carbon metabolism and thus DNA methylation [16, 17] correlates with the severity of Th2 immune responsiveness and allergic airway disease. The availability of methyl donors also associates with an altered T lymphocyte phenotype including skewing of the CD4^+^/CD8^+^ lymphocyte ratio, and production of CC chemokine and IL-4 (unpublished data). Naïve T lymphocyte differentiation into either Th1 or Th2 lineages is regulated through DNA methylation [37, 38], and CD4+ T cells have shown to have folate-sensitive regions [39]. Evaluation of neonates showed a correlation between high maternal folate levels and permissive epigenetic marks at the *GATA3* and *IL-9* loci in CD4+ T cells [40]. Previous *in vitro* studies have observed that hypomethylation in CD4^+^ T cells leads to increased expression of STAT4 and IFNγ [41] promoting a Th1 response and enhances FoxP3 expression [42] increasing the population of Treg cells. This could explain why we observe a reduced Th2 response in the hypomethylated HDM-treated C57BL/6^*Mthfr*-/-^ mice. Also, high levels of methyl donors lead to methylation and repression of *Runx3* (unpublished data), a key regulator of T lymphocyte development and promotor of Th1 cell lineage through the repression of IL-4 [43]. Furthermore, hypomethylation of *Runx3* is nominally significant (p-value = 0.002) in HDM-treated C57BL/6^*Mthfr*-/-^ compared to HDM-treated C57BL/6 mice which would support a more Th1 cell lineage. These findings indicate that Th2 immune responsiveness and development may be modifiable by single carbon metabolism.

Additionally, there are several possible explanations as to why a shift in immune response results from changes in DNA methylation and gene expression. Rasgrp4 is a guanine nucleotide exchange factor that activates Ras [44]. *Rasgrp4* maps to a region on chromosome 7 associated with baseline hyperreactivity in mice, and methacholine hyporesponsive C3H/HeJ mice express a dysfunctional Rasgrp4 protein [45]. *Rasgrp4* is highly expressed in mast cells [46], and loss of Rasgrp4 in mast cells leads to a reduction of FcεRI-mediated degranulation and cytokine production [47]. *Rasgrp4* is differentially methylated and expression is reduced in HDM-treated C57BL/6^MTHFR-/-^ mice which correlates with a blunted response upon challenge. Expression changes highlight immune signaling and developmental pathways providing additional evidence for changes in immune function. While the extent of methylation changes is modest, the genes differentially methylated could be master regulators leading to numerous downstream changes. For example, TLE4 is a co-repressor that binds with a number of DNA binding proteins leading to the recruitment of repressor elements. One repressor function of TLE4 is the inhibition of *IFNγ* through epigenetic mechanisms in Th2 cells in order to maintain Th2 identity [48]. TLE4 also alters PAX5, a B cell master regulator, from a transcriptional activator to a repressor [49]. Ingenuity upstream regulator analysis of differentially expressed genes between HDM-treated C57BL/6^*Mthfr*-/-^ and HDM-treated C57BL/6 mice predicted 9 out of the 21 DNA binding proteins that interact with Tle4 to be involved in the changes of expression (S6 Fig). This suggests that changes in key genes could account for numerous downstream changes. Nevertheless, the genes identified in this study are merely associations and further mechanistic work is needed to show direct relationship with the underlying biology that alters disease severity.

Polymorphisms of *MTHFR* appear to modulate a number of phenotypes. The C677T *MTHFR* SNP is common in the human population and affects global methylation [18]. It has been postulated that the C677T SNP may have immune-protective properties [50]. A study of hepatitis B virus (HBV) infection in a West African population showed that the T allele independently associated with the persistence of detectable anti-HBs antibodies and a reduced level of HBV DNA [51]. In another study, Mthfr deficient mice compared to wild-type littermates responded better to mouse cytomegalovirus infection in terms of early control of cytokine secretion, decreased viral titer, and spleen immune cell preservation [50]. Furthermore, a mouse model of cerebral malaria showed that *Mthfr* overexpression had worse survival rates compared to wild-type mice while *Mthfr* deficient mice survived longer [52]. Our results further demonstrate that loss of Mthfr function results in an altered immune response in a HDM model of allergic airway disease.

A limitation in this study is that the methylation experiment was completed on whole lung tissue with an admixture of cells. Cell type specific expression and methylation patterns increases variance in the analysis creating a higher threshold for discovery. Alternatively, the small number of methylation changes suggests that other regulatory mechanisms could be contributing to the shift in phenotype including histone modifications, non-coding RNAs, and/or protein modifications.

This is the first work to be published that has examined the role of Mthfr in an animal model of allergic airway disease. While the severe deficiency of Mthfr found in C57BL/6^*Mthfr*-/-^ mice is not common in the general population, our results may prove relevant to asthma in humans. Our approach in Mthfr deficient mice has identified genes that are regulated in disease and responsive to gene environment interactions. Furthermore, it highlights that asthma risk genes can be affected through both genetic and environmental mechanisms. For example, *TLE4* was previously associated with asthma in a GWAS with the associated SNP lying upstream of the gene likely affecting its regulation [53]. Here we have found an association between *Tle4* and allergic airway disease in mice through a change in DNA methylation suggesting that different paths likely lead to the development of allergic airway disease in mice.

## Supporting information

S1 Appendix Supplemental figure description. (DOCX)

S1 Fig INFγ ELISA. (TIF)

S2 Fig Expression network of immune signaling and developmental pathways. (TIF)

S3 Fig B129 SNP calls. (TIF)

S4 Fig Top 10 enriched IPA expression pathways. (TIF)

S5 Fig Expression and methylation development network. (TIF)

S6 Fig Tle4 interaction network. (TIF)

S1 Table Agilent SureSelect methyl sequencing summary. (XLSX)

S2 Table IPA enriched pathways from differentially expressed genes between the HDM-treated groups. (XLSX)

S3 Table Statistically significant DMRs in HDM-treated C57BL/6^MTHFR-/-^ and C57BL/6 mice. (XLSX)

S4 Table DMR subset analysis of gene lists. (XLSX)

S5 Table Significant methylation-expression correlations within 1MB. (XLSX)

S6 Table Pathways of significant methylation-expression correlations within 1MB. (XLSX)

S7 Table Pyrosequencing primers. (XLSX)
