# Supplementary material for: Methylene-tetrahydrofolate reductase contributes to allergic airway disease
Source: PLoS One. 2018 Jan 12;13(1):e0190916. doi: 10.1371/journal.pone.0190916 (PMC5766142; doi:10.1371/journal.pone.0190916)
Supplement: S1 Appendix — (DOCX) [file pone.0190916.s001.docx]

# Allergic Airway Disease is Dependent on Methylene-Tetrahydrofolate Reductase

Kenneth R Eyring, Brent S. Pedersen, Kenneth N. Maclean, Sally P. Stabler, Ivana V. Yang, David A. Schwartz

**Online Data Supplement**

**Supplemental Figure S1:** Inf-γ was observed in saline treated mice and was reduced or lost in HDM-treated mice indicating a shift toward Th2 upon HDM-treatment.

**Supplemental Figure S2:** A representative molecular network from IPA of differently expressed genes. Network analysis was performed using only direct interactions and a minimum network score of 20. Legend: genes are colored red (up-regulated) or green (down-regulated), horizontal ellipse = transcriptional regulator, square = cytokine, double circle = group/complex, upside down triangle = kinase, vertical diamond = enzyme, vertical rectangle = G-protein coupled receptor

**Supplemental Figure S3:** C57BL/6*^Mthfr^*^-/-^ sequence was compared against know B129 SNPs from the Mouse Genome Informatics database. Each dot represents a SNP plotted by its chromosomal location on the x-axis and the percentage of reads that matched on the y-axis.

**Supplemental Figure S4:** Subset analyses on methylation clusters between C57BL/6*^Mthfr^*^-/-^ and C57BL/6 mice located within 25kb of asthma related genes as defined by genetic association ([5](#_ENREF_5)), Ingenuity Pathway Analysis, genes included in the top 10 enriched IPA expression pathways from the study, or overlap of the 3 lists were performed. Statisically significant DMRs (adjusted p-value <0.10) were identified near *Tle4* and *Tnf*; *Scn5a*, *Pde7b*, *Tnf*, and *Rasgrp4*; *Nod2*; and *Tlr9* and *Tnf* respectively

**Supplemental Figure S5:** Methylation changes directly correlate with transcriptional activity. Methylation-expression relationships were measured by beta regression of differentially methylated regions (DMRs, uncorrected p-value <0.05, n = 6,927) associated with HDM-treated C57BL/6*^Mthfr^*^-/-^ mice and all expression probes found within 1Mb of each DMR. Ingenuity Pathway Analysis (IPA) on the 503 significant methylation-expression correlations (adjusted p-value <0.05) identified a significant inflammatory response network (score = 52). Green indicates lower methylation or expression and red indicates higher methylation or expression in HDM-treated C57BL/6*^Mthfr^*^-/-^ mice. Methylation values are colored based on relative methylation change between HDM-treated C57BL/6*^M^*^thfr-/-^ and HDM-treated C57BL/6 mice; colors of expression values are based on fold change between HDM-treated C57BL/6*^Mthfr^*^-/-^ and HDM-treated C57BL/6 mice. Molecule shapes: horizontal oval = transcriptional regulator; vertical oval = transmembrane receptor; diamond = enzyme; up triangle = phosphatase; down triangle = kinase; trapezoid = transporter; circle = other; double circle = group; rectangle = G-protein coupled receptor. This analysis was restricted to only direct relationships. The network score is based on the hypergeometric distribution, and is calculated with the right-tailed Fisher’s exact test to identify enrichment of correlated methylated/expressed genes in the network relative to IPA database.

**Supplemental Figure S6:** Ingenuity upstream analysis on differentially expressed genes between HDM-treated C57BL/6*^Mthfr^*^-/-^ and HDM-treated C57BL/6 mice predicted 9 (circled in red) of the 21 DNA binding proteins that interact with TLE4 to be involved in changes of expression.
