# Supplementary figures and images for: Methylene-tetrahydrofolate reductase contributes to allergic airway disease
Source: PLoS One. 2018 Jan 12;13(1):e0190916. doi: 10.1371/journal.pone.0190916 (PMC5766142; doi:10.1371/journal.pone.0190916)

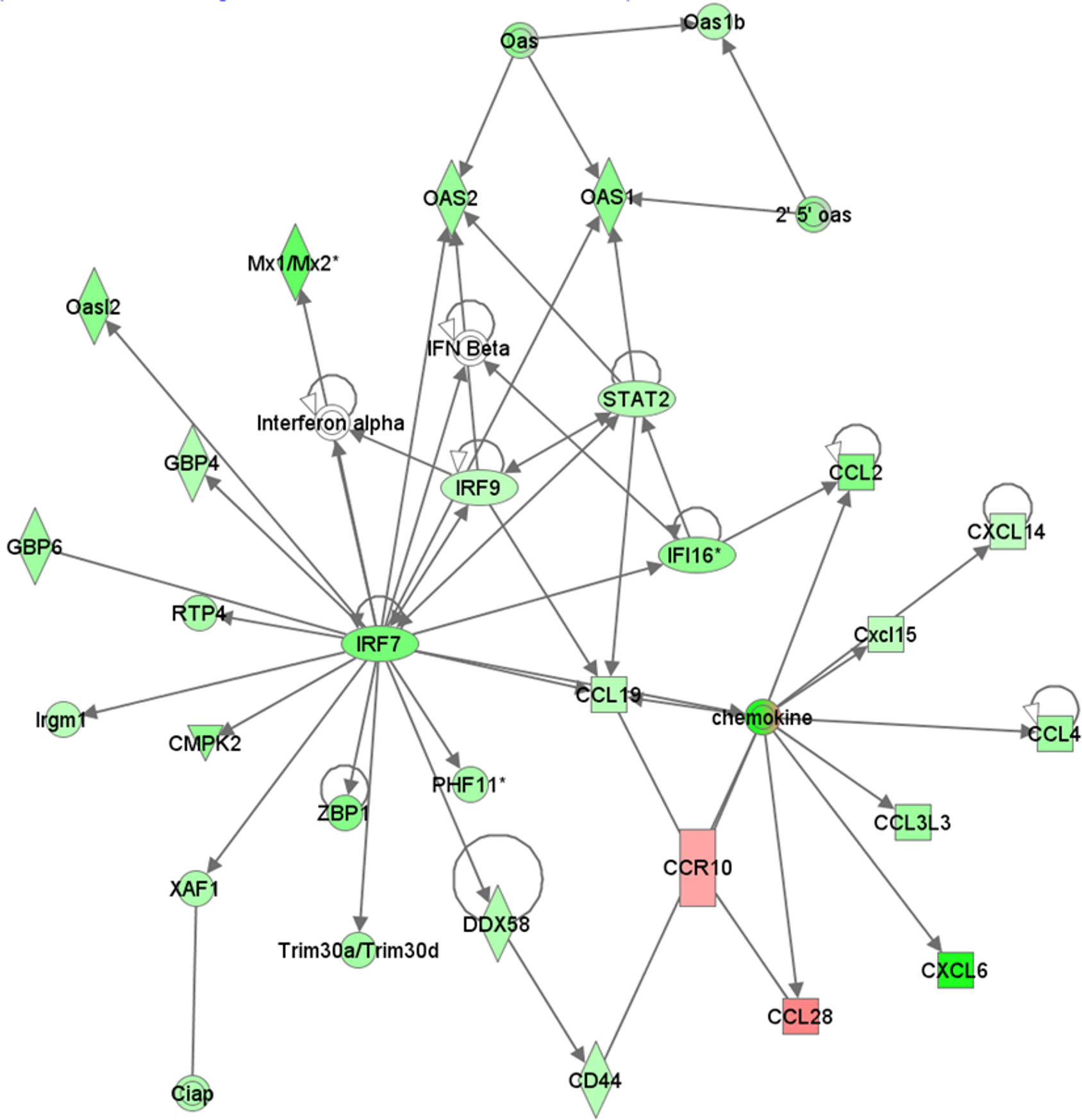

Supplement: S2 Fig — (TIF) [file pone.0190916.s003.tif]

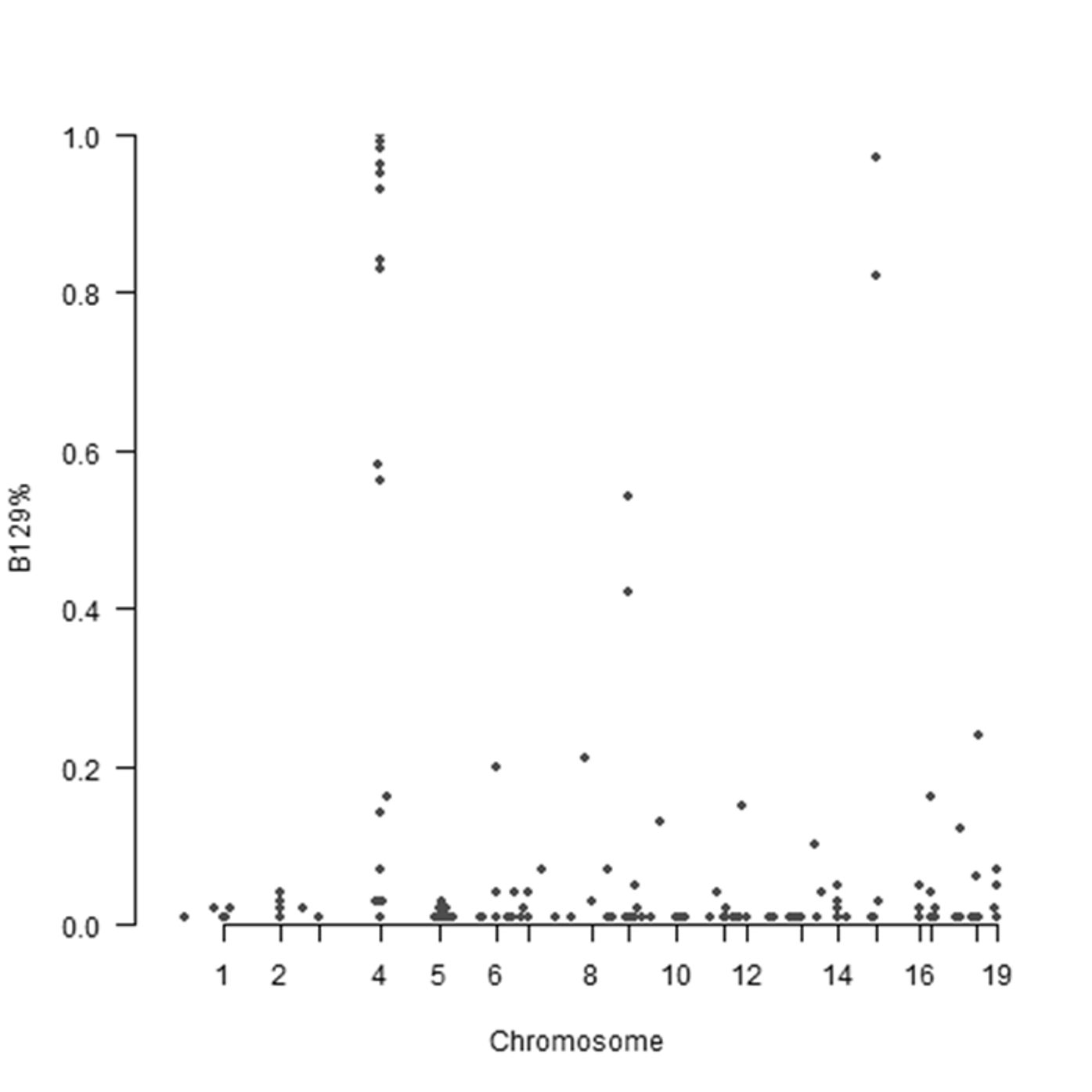

Supplement: S3 Fig — (TIF) [file pone.0190916.s004.tif]

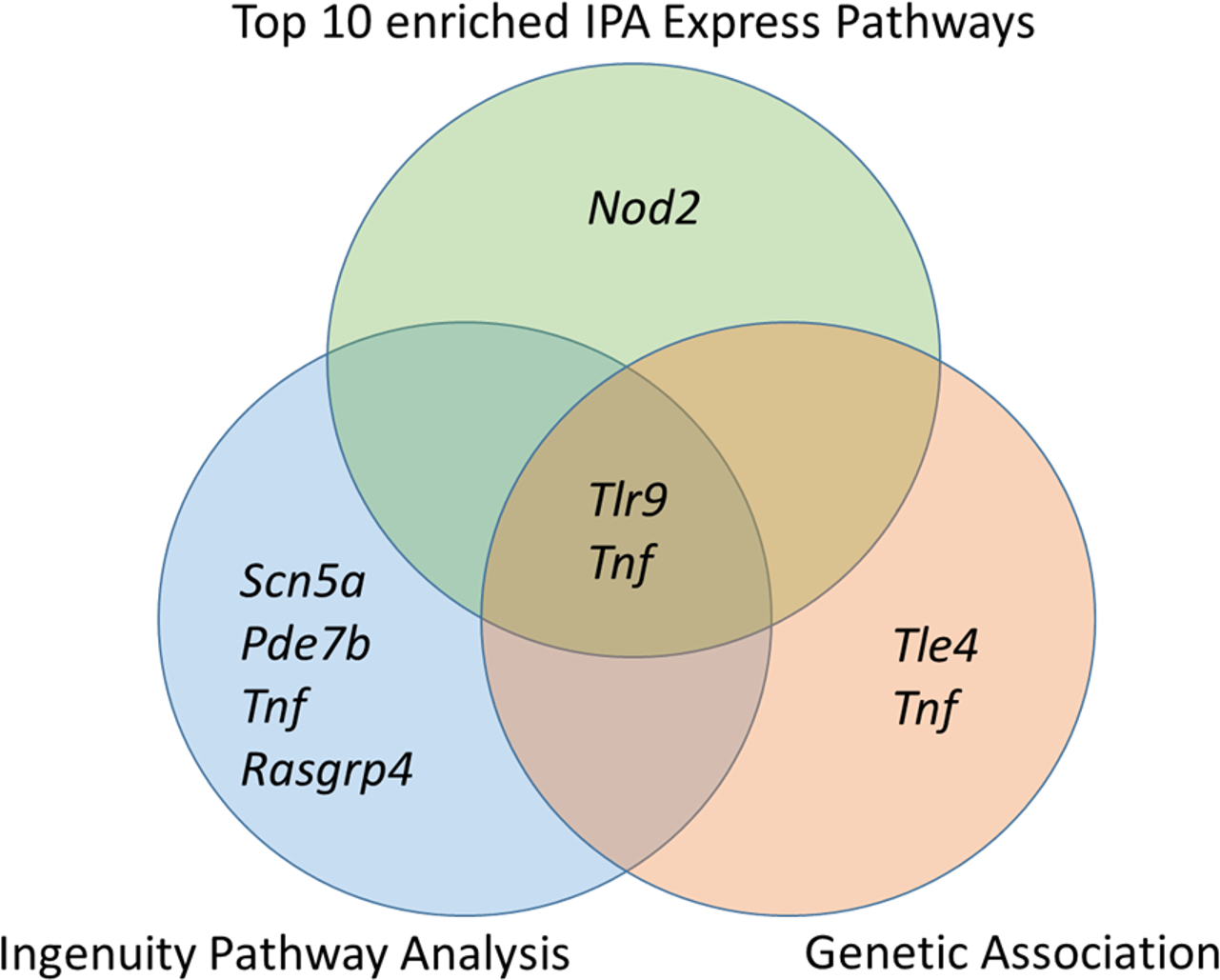

Supplement: S4 Fig — (TIF) [file pone.0190916.s005.tif]

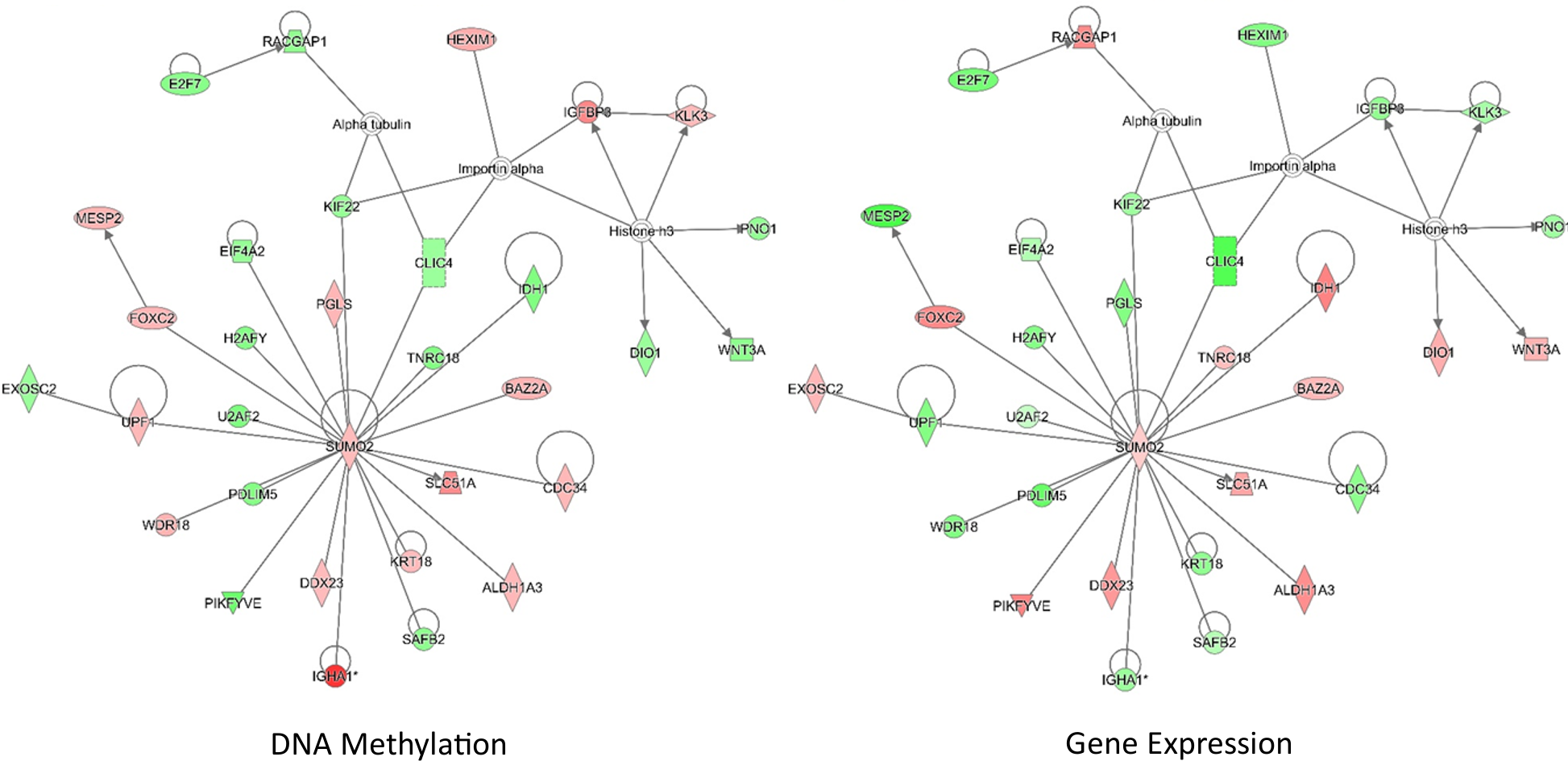

Supplement: S5 Fig — (TIF) [file pone.0190916.s006.tif]

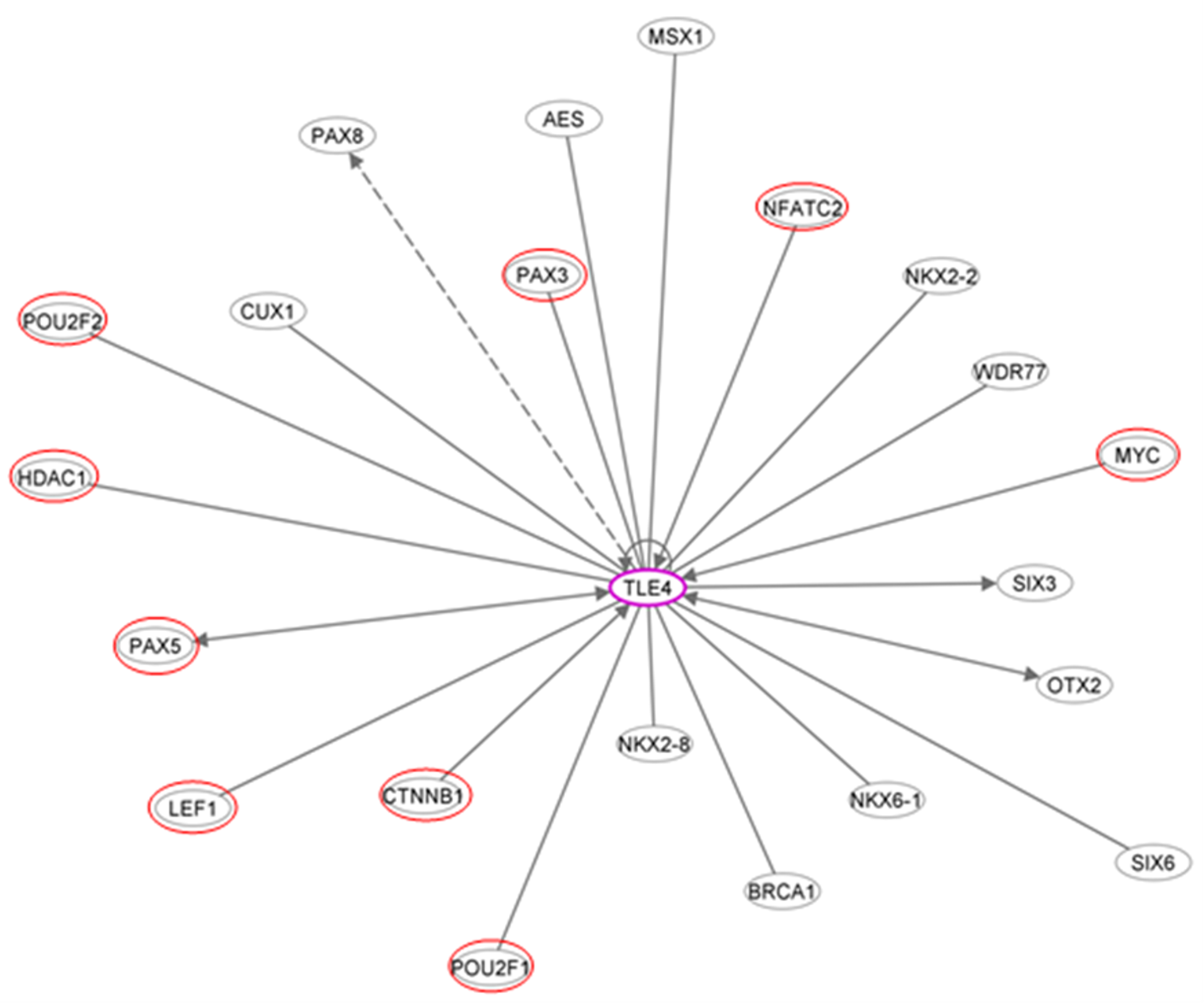

Supplement: S6 Fig — (TIF) [file pone.0190916.s007.tif]
